# Chronic NF-κB activation links COPD and lung cancer through generation of an immunosuppressive microenvironment in the lungs

**DOI:** 10.18632/oncotarget.6562

**Published:** 2015-12-11

**Authors:** Rinat Zaynagetdinov, Taylor P. Sherrill, Linda A. Gleaves, Pierre Hunt, Wei Han, Allyson G. McLoed, Jamie A. Saxon, Harikrishna Tanjore, Peter M. Gulleman, Lisa R. Young, Timothy S. Blackwell

**Affiliations:** ^1^ Department of Medicine, Division of Allergy, Pulmonary and Critical Care Medicine, Vanderbilt University School of Medicine, Nashville, TN, USA, 37232; ^2^ Department of Cancer Biology, Vanderbilt University, Nashville, TN, USA, 37232; ^3^ U.S. Department of Veterans Affairs, Nashville, TN, USA, 37232; ^4^ Division of Pulmonary Medicine, Department of Pediatrics, Vanderbilt University School of Medicine, Nashville, TN, USA, 37232

**Keywords:** NF-κB, COPD, lung cancer, macrophage, tregs

## Abstract

Nuclear Factor (NF)-κB is positioned to provide the interface between COPD and carcinogenesis through regulation of chronic inflammation in the lungs. Using a tetracycline-inducible transgenic mouse model that conditionally expresses activated IκB kinase β (IKKβ) in airway epithelium (IKTA), we found that sustained NF-κB signaling results in chronic inflammation and emphysema by 4 months. By 11 months of transgene activation, IKTA mice develop lung adenomas. Investigation of lung inflammation in IKTA mice revealed a substantial increase in M2-polarized macrophages and CD4^+^/CD25^+^/FoxP3^+^ regulatory T lymphocytes (Tregs). Depletion of alveolar macrophages in IKTA mice reduced Tregs, increased lung CD8^+^ lymphocytes, and reduced tumor numbers following treatment with the carcinogen urethane. Alveolar macrophages from IKTA mice supported increased generation of inducible Foxp3^+^ Tregs *ex vivo* through expression of TGFβ and IL-10. Targeting of TGFβ and IL-10 reduced the ability of alveolar macrophages from IKTA mice to induce Foxp3 expression on T cells. These studies indicate that sustained activation of NF-κB pathway links COPD and lung cancer through generation and maintenance of a pro-tumorigenic inflammatory environment consisting of alternatively activated macrophages and regulatory T cells.

## INTRODUCTION

Chronic obstructive pulmonary disease (COPD) is the 3^rd^ most common cause of death in the US [1] and is highly associated with an increased risk of lung cancer [2, 3]. After controlling for smoking history, patients with COPD have up to 6-fold increase in lung cancer incidence compared to smokers without COPD [4]. However, the mechanisms linking chronic airway inflammation and lung tumorigenesis remain unclear.

Current evidence suggests a role for NF-κB in COPD and lung cancer. Enhanced activation of canonical NF-ĸB signaling has been detected in airway epithelium of COPD patients [5] and in areas of dysplasia and premalignant lesions in biopsies from patients at risk for cancer [6]. In mice, activation of the NF-κB pathway has been detected during K-ras oncogene-driven lung adenocarcinoma [7, 8]. We have previously demonstrated that inhibition of NF-κB signaling in the airway epithelium significantly reduces formation of lung tumors [9]. In contrast, activation of NF-κB in the lungs markedly increases tumor formation [10], supporting the concept that activation of canonical NF-κB pathway plays an important role in lung carcinogenesis.

In these studies, we investigated whether NF-κB-dependent chronic inflammation could drive development of both COPD and lung tumors. We used a transgenic mouse model that conditionally expresses activated IκB kinase β (IKKβ) in airway epithelium (IKTA mice) [11]. We found that sustained activation of the NF-κB pathway in IKTA mice increases infiltration of lungs with M2-polarized macrophages and lymphocytes and results in both emphysema and small airway remodeling. With long-term transgene activation, NF-κB-mediated chronic airway inflammation supports development of spontaneous lung tumors. In additional studies, we found that alveolar macrophages are essential for increased lung tumorigenesis following treatment with the carcinogen urethane. In the context of NF-κB-driven chronic airway inflammation, macrophages support carcinogenesis through TGFβ/IL-10/retinoic acid-dependent mechanisms that enhance generation of immunosuppressive regulatory T cells (Tregs).

## RESULTS

### Persistent activation of the NF-κB pathway in airway epithelium results in emphysema, small airway remodeling, and spontaneous tumor formation

**Figure 1:**
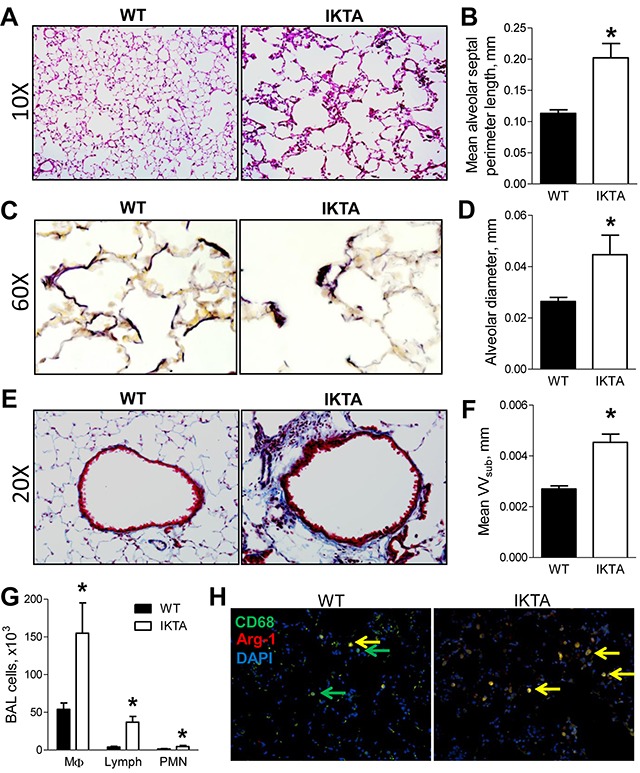
Sustained NF-κB activation in airway epithelium results in airway remodeling and emphysema **A.** Representative photomicrographs of H&E stained lung sections, **B.** mean alveolar septal perimeter length per lung section, **C.** representative photomicrographs of elastin staining using Verhoeff's Van Gieson protocol, and **D.** mean alveolar diameter from lungs of WT and IKTA mice treated with dox (0.1 g/L) for 4 months (n = 6 mice per group, *p < 0.05). **E.** Representative photomicrographs of Masson's-Trichrome stained lung sections and **F.** morphometric evaluation of volume density (VVsub) of subepithelial connective tissue as a measure of airway wall thickness in small airways (<2 mm) from WT and IKTA mice at 4 months of treatment with dox (n = 6 mice per group, *p < 0.05). **G.** BAL macrophages (MΦ), lymphocytes (lymph), and neutrophils (PMN) from WT and IKTA mice at 4 months of treatment with dox (n = 6 mice per group). Data are presented as mean ± SEM, *p < 0.05. **H.** Immunostaining for CD68 (green), arginase-1 (red), and DAPI to detect M2 macrophages (yellow) in lungs of WT and IKTA mice at 4 months of treatment with dox.

To investigate the effects of long-term NF-κB-mediated inflammation in the lungs, we first treated IKTA mice and WT littermate controls with doxycycline (dox, 0.1g/L in drinking water) for 4 months. Dox-treated IKTA mice developed diffuse emphysematous enlargement of distal airspaces compared to dox-treated WT controls, which was evident by histologic evaluation (Figure 1A) and morphometric determination of alveolar septal perimeter length and alveolar diameter (Figure 1B and 1D). In comparison, no differences were detected in alveolar size/appearance in WT and IKTA mice that were not treated with dox (data not shown). As opposed to the linear appearance of elastin fibers in the alveolar compartment of WT mice, the elastin network in dox-treated IKTA mice appeared disorganized and clumped at the tips of alveolar ducts and loose/unraveled in inter-alveolar walls (Figure 1C), as has been documented in human emphysema [12]. In addition to emphysema, fibrotic thickening of the walls of small airways is one of the central features of COPD [3]. Therefore, we analyzed airway wall thickness (subepithelial connective tissue volume density, VV_sub_) in small airways from the lungs of dox-treated WT and IKTA mice. This morphometric analysis showed a marked increase in airway wall thickness in dox-treated IKTA mice at 4 months (Figures 1E and 1F). In addition, we found significantly increased numbers of macrophages, lymphocytes, and neutrophils in bronchoalveolar lavage (BAL) (Figure 1G). To determine the phenotype of lung macrophages in IKTA mice at this time point, we immunostained lung sections from WT and IKTA mice with anti-arginase-1 and anti-CD68 antibodies to detect M2-polarized macrophages. As shown in Figure 1H, we observed a marked increase in arginase-1+/CD68+ macrophages in the lungs of IKTA mice compared with controls.

**Figure 2:**
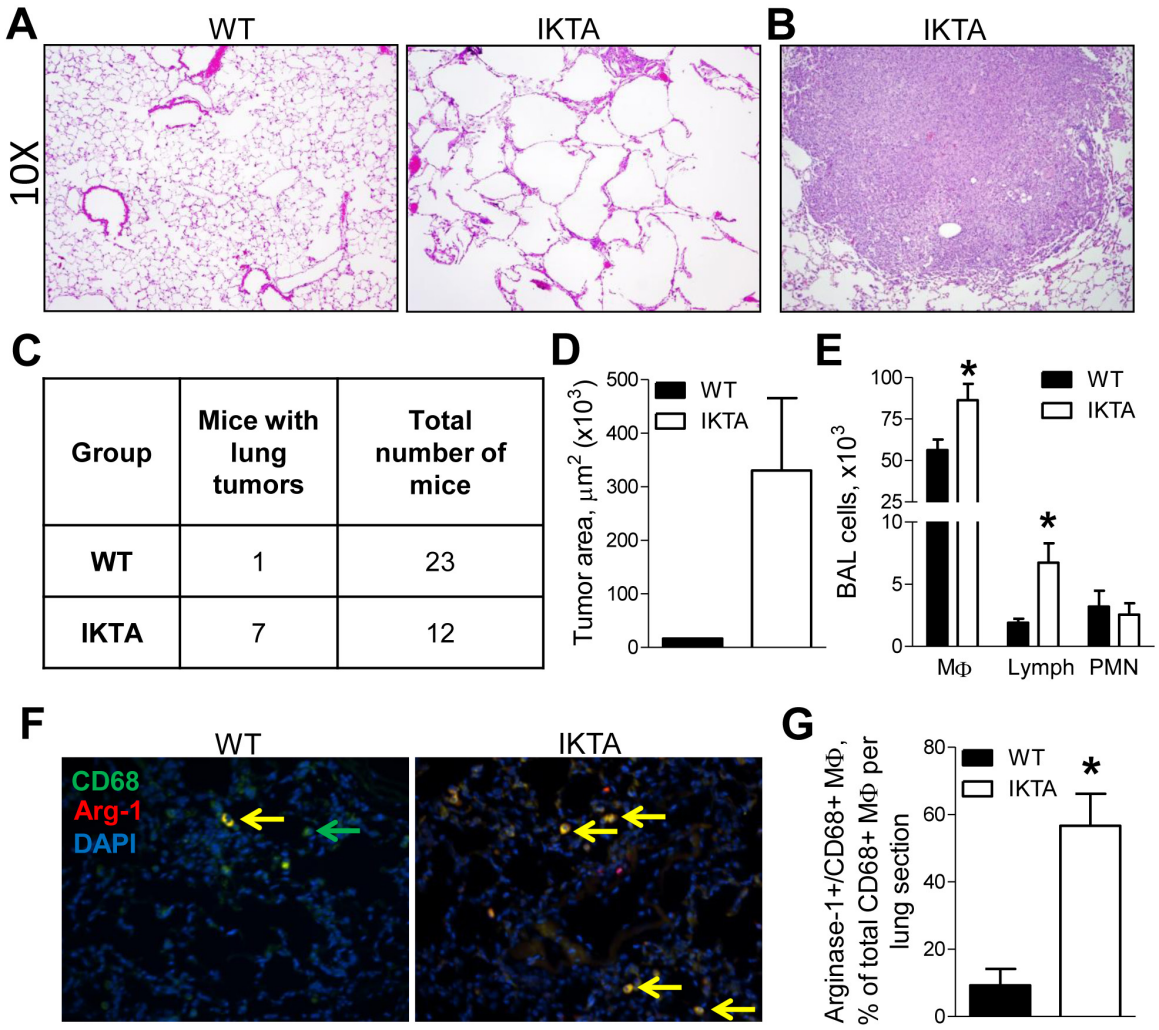
NF-κB-induced chronic airway inflammation induces spontaneous development of lung tumors Representative photomicrographs of **A.** H&E stained lung sections from WT and IKTA mice and **B.** lung tumor (10X magnification) in IKTA mouse treated with dox (0.1 g/L) for 11 months. **C.** Table showing numbers of tumor-bearing mice and **D.** area of lung tumors in WT and IKTA mice after continuous treatment with dox (0.1 g/L) for 11 months. **E.** BAL macrophages (MΦ), lymphocytes (Lymph), and neutrophils (PMN) from WT and IKTA mice. **F.** Representative photomicrographs of immunostaining for CD68 (green), arginase-1 (red), and DAPI to detect M2 macrophages (yellow) and **G.** the number of Arginase-1+/CD68+ macrophages per lung sections from WT and IKTA mice after continuous treatment with dox (0.1 g/L) for 11 months. *p < 0.05.

To further investigate the consequences of long-term NF-κB activation in the lungs, we treated IKTA mice and WT littermate controls with continuous dox for 11 months. In addition to substantial COPD-like lung remodeling, 7/12 IKTA mice developed spontaneous lung tumors that had the appearance of large, solid adenomas. In contrast, only 1/23 WT control mice developed a single small lung tumor (Figure 2A–2D). As expected, IKTA mice had increased numbers of macrophages and lymphocytes in bronchoalveolar lavage (Figure 2E). We also observed a marked increase in arginase-1+/CD68+ macrophages in the lungs of IKTA mice compared with controls (Figure 2F–2G). These findings indicate that sustained NF-κB activation in airway epithelium results in COPD-like pathology followed by induction of lung tumorigenesis, illustrating a mechanistic link between COPD and lung cancer via the NF-κB pathway.

### Alveolar macrophages in IKTA mice are predominantly M2-polarized and possess immunosuppressive properties

**Figure 3:**
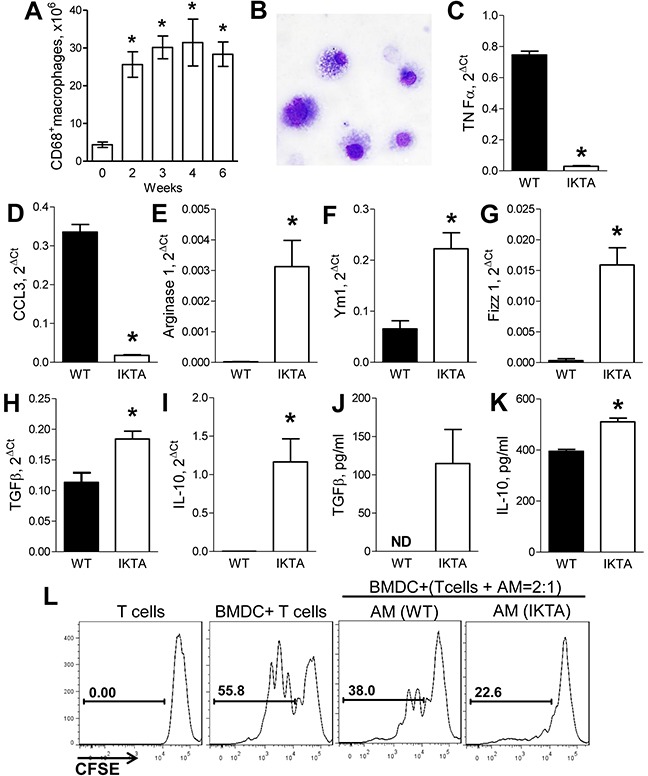
Accumulation of M2-polarized alveolar macrophages during NF-κB-induced chronic airway inflammation generates immunosuppressive microenvironment **A.** Number of CD45+/CD68+ macrophages identified by flow cytometry in lungs of IKTA mice at baseline (week 0) or after dox treatment (0.1 g/L, n = 3-6 mice per group per time point, *p < 0.05 compared to the baseline). **B.** Representative photomicrograph of alveolar macrophages collected from IKTA after 3 weeks of dox treatment. **C–I.** mRNA expression for M1 markers (TNFα, CCL3) and M2 markers (Ym1, Fizz1, Arginase-1, TGFβ, IL-10) by alveolar macrophages isolated from WT and IKTA mice after 3 weeks of continuous treatment with dox. *p < 0.05. **J.** Total TGFβ concentration in BAL and **K.** IL-10 concentration in whole lung homogenates collected from WT and IKTA mice after 3 weeks of dox treatment. ND - not detected, *p < 0.05. **L.** CD11b^+^ lung macrophages (MΦ) collected from WT and IKTA mice after 3 weeks of dox treatment reduce proliferation of CD4^+^ T cells stimulated by allogeneic bone marrow-derived dendritic cells (DC).

To further investigate macrophages in IKTA mice, we analyzed expression of M1 and M2 markers by lung macrophages isolated from IKTA or WT control mice at an earlier time point (after 3 weeks of dox). Our previous studies have shown that by this time point, the initial neutrophilic acute airway inflammation in IKTA mice shifts toward chronic inflammation with predominance of lymphocytes and macrophages [10], and the numbers of lung macrophages reach maximal values (Figure 3A). For these studies, we isolated macrophages from dox-treated IKTA mice or WT controls by BAL followed by enrichment using CD11b magnetic beads and 2-hour adherence to tissue culture plates. Purity of lung macrophages was confirmed by morphological examination of the cells (Figure 3B). Using quantitative RT-PCR, we found that lung macrophages from IKTA mice had significantly lower mRNA expression of the M1 markers TNFα and CCL3 compared to WT controls (Figures 3C–3D). We did not detect significant expression of iNOS by lung macrophages from IKTA or WT mice (data not shown). In contrast, mRNA expression of M2 markers (Ym1, Fizz1, Arginase-1, TGFβ, and IL-10) by macrophages from IKTA mice was significantly higher than WT macrophages (Figures 3E–3I). Increased expression of TGFβ and IL-10 in lungs of IKTA mice was confirmed by ELISA (Figure 3J–3K). Together, these findings indicate that persistent NF-κB activation in the airway epithelium drives M2 polarization of alveolar macrophages as a component of chronic airway inflammation.

Since suppression of anti-tumor response has been recognized as one of the major mechanisms by which myeloid cells can contribute to tumor promotion, we evaluated whether lung macrophages isolated from IKTA and WT mice after 3 weeks of dox treatment possess immunosuppressive properties. We performed an allogeneic mixed leukocyte reaction (MLR) assay in which CD4^+^/CD25^−^ effector T cells (Teff) from spleens of untreated WT mice were activated with allogeneic dendritic cells and co-cultured with alveolar macrophages purified from lungs of dox-treated WT or IKTA mice as described above. Proliferation of effector T cells was evaluated after 3 days of co-culture by measuring dilution of carboxyfluorescein succinimidyl ester (CFSE) fluorescence. We found that alveolar macrophages from WT or IKTA mice were not able to induce proliferation of effector T cells (data not shown); rather, these cells reduced proliferation of T cells stimulated with allogeneic DCs (Figure 3L). We also found that alveolar macrophages from IKTA mice caused more profound impairment of T cell proliferation compared with macrophages from WT mice. These findings suggest that M2-polarized alveolar macrophages from IKTA mice support an immunosuppressive microenvironment in the distal lung.

### Alveolar macrophages play a pivotal role in promotion of lung tumors in the context of chronic airway inflammation

**Figure 4:**
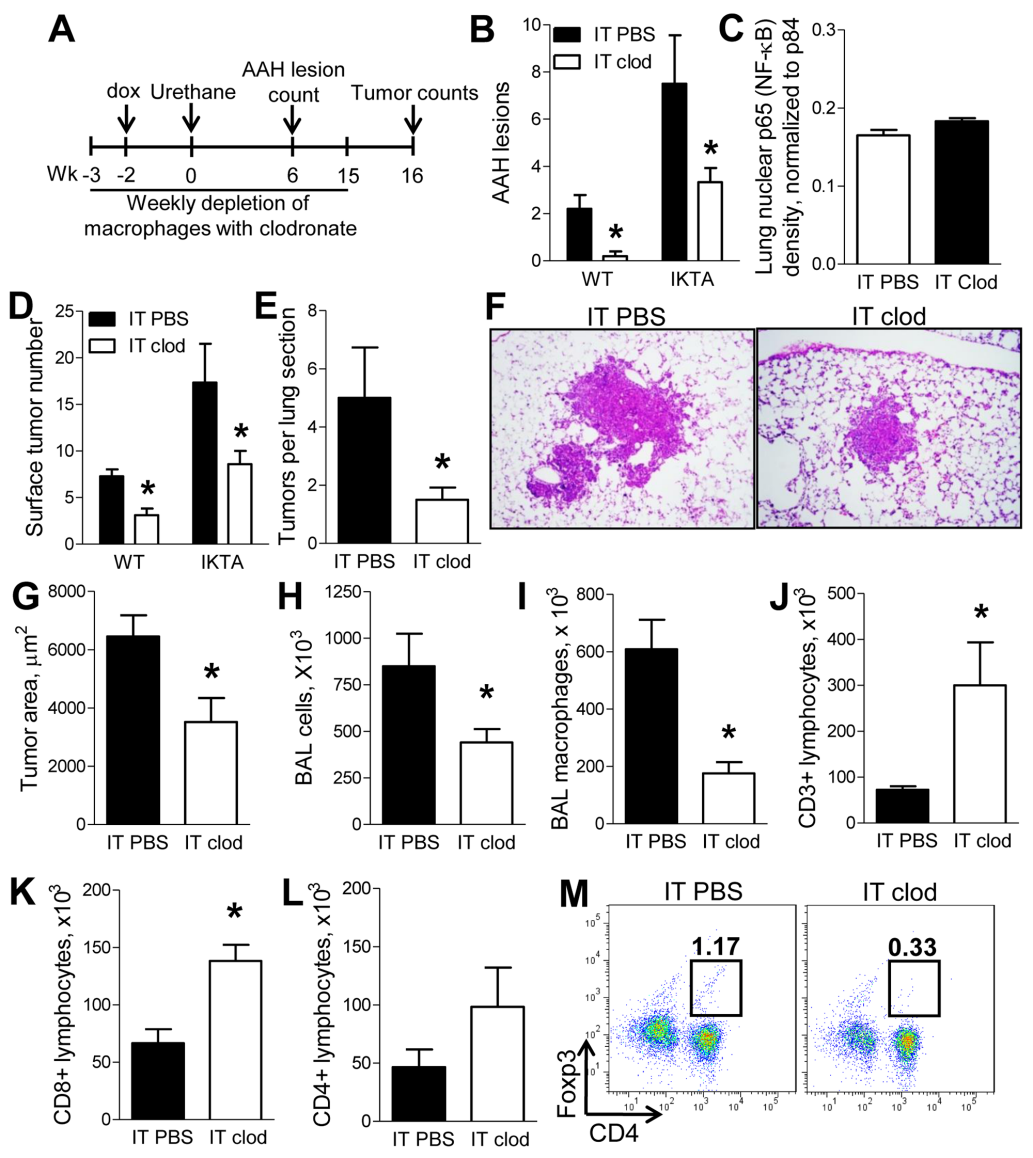
Alveolar macrophages support enhanced lung carcinogenesis during NF-κB-mediated chronic airway inflammation **A.** Schematic representation of macrophage depletion experiments in dox-treated WT and IKTA mice treated with urethane by intraperitoneal (IP) injection. **B.** Mean number of AAH lesions for each mouse counted per H&E-stained lung section from dox-treated WT and IKTA mice treated weekly with intratracheal (IT) injections of control (PBS) liposomes or clodronate liposomes and harvested at week 6 after IP injection of urethane (n = 6-9 mice per group, *p < 0.05 compared to PBS liposome group). **C.** Densitometry analysis of western blots for p65 (RelA) normalized for p84 expression in lung nuclear protein extracts from IKTA mice treated with weekly IT injections of PBS liposomes or clodronate liposomes for 6 weeks after IP urethane injection (n = 4 mice per group, *p < 0.05 compared to PBS liposome group). **D.** Numbers of lung surface tumors, **E.** tumors per H&E-stained lung section, **F.** representative photomicrographs of lung tumors, and **G.** mean tumor size from lungs of dox-treated WT and IKTA mice treated weekly with PBS liposomes or clodronate liposomes and harvested at 4 months after urethane injection (n = 6-9 mice per group, *p < 0.05 compared to PBS liposome group). **H.** Total BAL cells and **I.** macrophages from dox-treated IKTA mice treated weekly with PBS or clodronate liposomes for 4 months after urethane injection. **J–L.** Total CD3+ lymphocytes, CD8+ lymphocytes, CD4+ lymphocytes, and **M.** percentage of CD4+/Foxp3+ Tregs identified by flow cytometry in lungs of dox-treated IKTA mice treated weekly with PBS or clodronate liposomes and analyzed at 4 months after urethane injection (n = 4-9 mice per group, *p < 0.05).

In order to investigate the factors that contribute to a pro-tumorigenic environment in the setting of NF-κB-generated chronic airway inflammation, we employed the urethane model of lung carcinogenesis. In this model, we depleted alveolar macrophages using a previously described protocol [10] with weekly intratracheal (IT) injections of liposomal clodronate. As depicted in Figure 4A, IKTA mice and WT control mice were treated with weekly IT injections of liposomal clodronate (or PBS-containing liposomes) followed by dox treatment and a single intraperitoneal (IP) injection of urethane. Consistent with previous findings [13], depletion of alveolar macrophages with clodronate in WT mice significantly reduced the number of premalignant atypical adenomatous hyperplasia (AAH) lesions in the lungs by week 6 after urethane (Figure 4B). In IKTA mice, macrophage depletion reduced AAH lesions to numbers similar to urethane-injected WT mice treated with control “PBS” liposomes (Figure 4B), suggesting that during chronic airway inflammation alveolar macrophages could be responsible for enhanced early formation of lung tumors. To determine whether depletion of alveolar macrophages altered NF-κB activity in the lungs of IKTA mice treated with PBS- or clodronate-liposomes for 6 weeks, we performed western blot analysis for nuclear p65 (RelA) and found no differences in NF-κB activity following macrophage depletion (Figure 4C). Next, we performed macrophage depletion studies in WT and IKTA mice as described in Figure 4A and assessed lung tumors at 4 months (16 weeks) after urethane injection. We found that depletion of alveolar macrophages significantly reduced tumor formation in WT and IKTA mice (Figure 4D). Evaluation of lung sections from IKTA mice confirmed these findings (Figure 4E). In addition, we found that depletion of alveolar macrophages with clodronate reduced the size of lung tumors in IKTA mice (Figure 4F–4G). Interestingly, we did not detect a reduction in emphysema in lungs of clodronate or control (PBS) liposome-treated IKTA mice at this time point. The mean alveolar septal perimeter length in PBS and clodronate liposome-treated IKTA mice was 0.18±0.01 mm compared to 0.17±0.01 mm, respectively (p = 0.21). As expected, treatment of IKTA mice with liposomal clodronate significantly reduced BAL macrophage numbers (Figure 4H, 4I). To better characterize changes in other lung leukocyte subsets in urethane-injected IKTA mice after depletion of alveolar macrophages, we performed flow cytometric analysis. Depletion of alveolar macrophages resulted in a significant increase in CD3+ lung lymphocytes (Figure 4J). Analysis of lymphocyte subsets in clodronate-treated IKTA mice revealed increased CD8^+^ lymphocytes (Figure 4K), but no differences in total CD4+ lymphocytes (p = 0.24, Figure 4L). However, depletion of alveolar macrophages markedly reduced the subpopulation of CD4^+^/Foxp3^+^ regulatory T lymphocytes (Tregs) (Figure 4M). Together, these findings are consistent with our prior report that Tregs are important for enhanced tumorigenesis in IKTA mice [10] and suggest that alveolar macrophages are responsible for increased generation and/or recruitment of these cells.

### During chronic airway inflammation, alveolar macrophages create an immunosuppressive pro-tumorigenic microenvironment by inducing the generation of Foxp3+ Tregs

**Figure 5:**
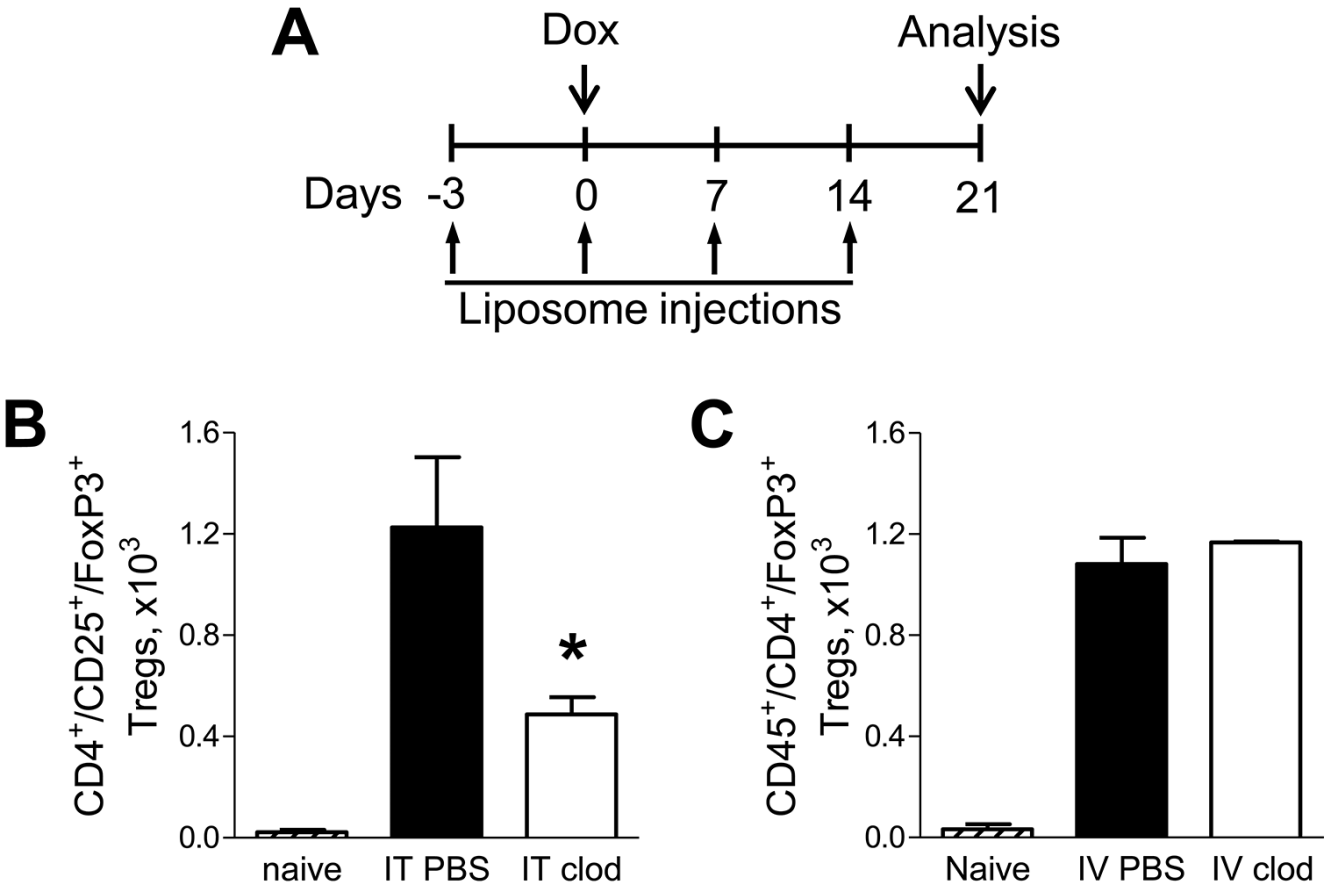
Alveolar macrophages support increased infiltration of lungs with Foxp3^+^ Tregs during chronic inflammation **A.** Schematic representation of macrophage depletion strategy in IKTA mice. Numbers of viable CD45^+^/CD4^+^/CD25^+^/Foxp3^+^ Tregs in lungs of naïve (untreated) or dox-treated IKTA mice that received **B.** IT clodronate (IT clod) to deplete alveolar macrophages, or **C.** IV clodronate (IV clod) to deplete interstitial macrophages. IT or IV PBS-liposome injections used as a control (n = 3-4 mice per group, *p < 0.05 compared to PBS-liposome group).

To prove that alveolar macrophages contribute to increased infiltration of the lungs with Tregs in the setting of NF-κB-generated chronic inflammation, we treated IKTA mice with dox and IT injections of liposomal clodronate on days −3, 0, 7, and 14 relative to dox treatment to deplete alveolar macrophages (Figure 5A). Dox-treated IKTA mice injected with control (PBS) liposomes or naïve IKTA mice (no dox or liposome injections) were used as controls. Analysis of lung Tregs was conducted at day 21 (week 3) using flow cytometry. As expected, activation of airway NF-κB signaling markedly increased Tregs in the lungs of IKTA mice treated with control (PBS) liposomes; however, Tregs were significantly reduced after depletion of alveolar macrophages with clodronate liposomes (Figure 5B).

Treatment of mice with liposomal clodronate through the IT route efficiently depletes alveolar macrophages but has minimal impact on interstitial macrophages [14, 15]. To investigate whether interstitial macrophages could also contribute to Treg accumulation in the lungs during chronic airway inflammation, we targeted these cells for depletion by intravenous (IV) injection of liposomal clodronate [16]. We treated IKTA mice with clodronate liposomes or PBS liposomes through tail vein injections and found that depletion of interstitial macrophages did not alter the Treg population in the lungs of dox-treated IKTA mice (Figure 5C).

**Figure 6:**
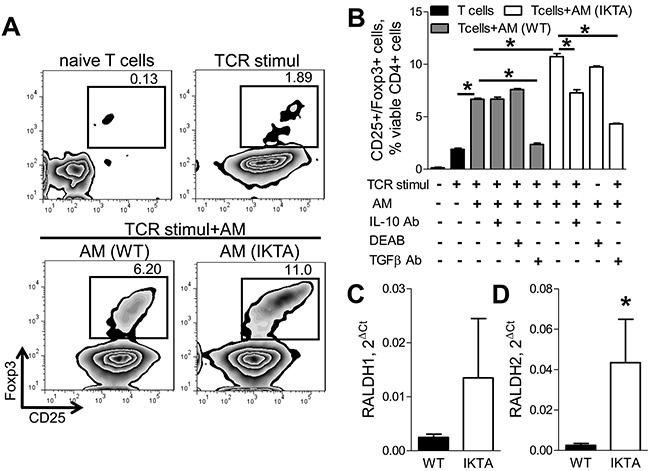
Alveolar macrophages induce generation of Foxp3^+^ Tregs through production of TGFβ, IL-10, and retinoic acids during chronic airway inflammation **A.** Representative FACS plots and **B.** CD4^+^/CD25^+^/Foxp3^+^ Tregs generated *ex vivo* by syngeneic alveolar macrophages (AM) from naive CD4+^−^ T cells (T cell: AM ratio is 2:1) alone or after treatment with antibodies to TGFβ (10 ug/ml, 1D11) or IL-10 (10 ug/ml, JES5-2A5), or in presence of an inhibitor of retinoic acid synthesis (DEAB, 15 uM). Alveolar macrophages were collected from WT and IKTA mice after 3 weeks of continuous treatment with dox (0.1g/L). Prior to co-culture CD4+ naive T cells were activated through T cell receptors (TCR stimul) using CD3/CD28 antibody cocktail. Analysis of CD4+CD25+Foxp3+ Tregs was performed in triplicate after 3 days of co-culture, *p < 0.05. **C–D.** mRNA expression of RALDH1 and RALDH2 by alveolar macrophages from WT and IKTA mice after 3 weeks of continuous treatment with dox (0.1g/L). *p < 0.05.

Since alveolar macrophages affect Treg accumulation in the lungs, we tested whether macrophages support increased Foxp3^+^ Treg generation from naïve T lymphocytes. We performed *in vitro* studies in which naïve CD4+ T cells from untreated WT mice were activated with CD3/CD28 antibodies and co-cultured with syngeneic alveolar macrophages isolated from either IKTA or WT mice after 3 weeks of dox. Analysis of viable CD4^+^/CD25^+^/Foxp3^+^ Tregs was performed after 3 days of T cell/alveolar macrophage co-culture using flow cytometry. Although we found that alveolar macrophages from both dox-treated WT and IKTA mice induced generation of CD4^+^/CD25^+^/Foxp3^+^ Tregs *ex vivo*, co-culture with alveolar macrophages from dox-treated IKTA mice resulted in significantly more CD4^+^/CD25^−^/Foxp3^+^ Tregs compared to WT alveolar macrophages (Figure 6A).

Since alveolar macrophages from IKTA mice had significantly elevated TGFβ and IL-10 expression (Figure 3H–3K), both of which have also been previously reported to induce Foxp3 expression [17, 18], we evaluated whether alveolar macrophages from IKTA mice could induce Treg differentiation through a TGFβ/IL-10-dependent mechanism. We tested whether blocking TGFβ or IL-10 signaling using specific antibodies could reduce generation of Foxp3+ Tregs by alveolar macrophages *ex vivo*. In this study, naïve CD4+ T cells isolated from spleens of WT mice were stimulated through CD3 and CD28 and co-cultured with alveolar macrophages from IKTA mice (collected after 3 weeks of continuous dox treatment) in the presence of TGFβ or IL-10 neutralizing antibodies or specific isotype control antibodies. We found that treatment with anti-IL-10 antibodies (10μg/ml) caused partial (30%) reduction in the number of CD4^+^/CD25^+^/Foxp3^+^ Tregs after co-culture with alveolar macrophages from IKTA mice but not with macrophages from WT mice (Figures 6B). At the same time, TGFβ-neutralizing antibodies markedly diminished CD4^+^/CD25^+^/Foxp3^+^ Tregs after co-culture with alveolar macrophages from both dox-treated WT and IKTA mice (Figures 6B).

Retinoic acids have also been shown to contribute to conversion of naïve T cells into Foxp3+ Tregs [17, 19]. Therefore, we next assessed expression of key enzymes for the synthesis of retinoic acids RALDH1 and RALDH2 [19] by alveolar macrophages collected from WT and IKTA mice. While we did not detect significant differences in expression of RALDH1 (Figure 6C), alveolar macrophages from IKTA mice had significantly increased expression of RALDH2 (Figures 6D). Next, we tested whether retinoic acids contribute to generation of Foxp3+ Tregs by alveolar macrophages from IKTA mice *ex vivo*. We performed co-culture studies as described above in the presence of a RALDH inhibitor (diethylaminobenzaldehyde, DEAB, 15 μM) [20]. The number of viable CD4^+^/CD25^+^/Foxp3^+^ Tregs was assessed by flow cytometry after 3 days of co-culture. As shown in Figure 6B, inhibition of retinoic acid synthesis with DEAB caused modest but statistically significant (p = 0.03) reduction in numbers of Foxp3^+^ Tregs generated by alveolar macrophages from IKTA, but not WT mice. Importantly, treatment of macrophages for 3 days with DEAB did not affect viability of alveolar macrophages *ex vivo* (data not shown). Together, these findings suggest that alveolar macrophages can enhance lung tumorigenesis through TGFβ, retinoic acid, and IL-10-dependent immune suppression and generation of Tregs.

## DISCUSSION

While pro-inflammatory pathway activation is normally terminated after elimination of triggering stimuli, unresolved inflammation can lead to development of chronic inflammatory diseases, including COPD. In the current study, we demonstrate that sustained activation of NF-κB signaling links COPD-like pathology and lung cancer. We found that persistent activation of NF-κB signaling in airway epithelium results in chronic airway inflammation, small airway remodeling, and diffuse lung emphysema, similar to patients with COPD. We also found that NF-κB-driven chronic airway inflammation results in accumulation of M2-polarized macrophages, which support lung tumorigenesis by differentiating Tregs and down-regulating of anti-tumor CD8+ T lymphocytes. In addition, we demonstrated that elevated expression of TGFβ and IL-10 by alveolar macrophages contributes to the development and maintenance of an immunosuppressive microenvironment in the lungs despite the presence of chronic airway inflammation.

In COPD, activation of the NF-κB pathway has been detected in the bronchial epithelium and macrophages [5, 21]. In animal studies, activation of NF-κB signaling has been implicated in COPD-like inflammation and pathology induced by *Nontypeable Haemophilus influenzae* (NTHi) [22] and tobacco smoke [23]. Here, we demonstrated that sustained activation of the NF-κB pathway in airway epithelium causes chronic airway inflammation with increased infiltration of lungs with macrophages, lymphocytes, and neutrophils, along with fibrotic thickening of small airway walls and profound emphysema, strongly supporting a role for NF-κB signaling in COPD. Although additional work will be required to determine the proximate cause of emphysematous destruction in our model, alveolar macrophages do not appear to be critical since macrophage depletion did not substantially ameliorate this phenotype.

These are the first studies to show that NF-κB-induced chronic inflammation is sufficient to support tumorigenesis. Previously, NF-κB activation has been identified in premalignant lesions in humans and lung tumors in humans and mice [6–8, 24]. We showed that expression of a dominant NF-κB inhibitor in airway epithelium markedly reduced lung tumorigenesis in mice [9], while over-expression of an NF-κB activator in IKTA mice enhanced carcinogen-induced lung tumors and was able to function as a tumor promoter in the presence of an initiating agent (mutagen) [10]. These studies add to existing knowledge by showing that NF-κB activation can underlie both COPD-like lung remodeling and lung cancer and by demonstrating the oncogenic potential of persistent NF-κB-generated inflammation.

Alveolar macrophages appear to be critical players in promotion of lung tumors during chronic airway inflammation through regulation of the immune microenvironment. Our studies indicate that during chronic airway inflammation, alveolar macrophages are predominantly M2-polarized and appear to support tumorigenesis at least in part through generation of regulatory T cells. Elevated numbers of Tregs have been detected in lungs of patients with COPD [25]; however, the role of Tregs in COPD progression remains uncertain. In lung cancer, accumulation of Tregs correlates with poor clinical outcome [26]. Our findings are consistent with our previous demonstration that depletion of Tregs restores CD8^+^ T cell-mediated anti-tumor immune response and reduces lung carcinogenesis in IKTA mice [10]. Thus, during chronic airway inflammation, alveolar macrophages can promote lung tumorigenesis through generation of immunosuppressive Tregs.

After investigating the mechanisms by which alveolar macrophages regulate Treg generation, we found elevated expression of TGFβ by alveolar macrophages exposed to chronic inflammatory stimuli. Blocking TGFβ signaling with antibodies reduced the numbers of Tregs, consistent with prior reports that TGFβ regulates expression of Foxp3 [17]. We also observed a reduction in Tregs after targeting IL-10 or retinoic acids; however, this reduction was less pronounced compared to targeting TGFβ. Therefore, we suggest that during chronic inflammation, TGFβ, IL-10, and retinoic acids may function together to maintain an immunosuppressive, tumor-promoting microenvironment. Our findings are consistent with a report by Murai and colleagues showing that secretion of IL-10 by myeloid cells helps maintain Foxp3+ Tregs in a model of colitis [18].

While retinoic acids have been shown to support expression of Foxp3 in Tregs [19], blocking of retinoic acid production produced modest effects in our model. We speculate that retinoic acids could be more important for stability of Foxp3 expression in inducible Tregs, rather than for induction of Foxp3 expression per se. Despite reports of anti-tumor properties of vitamin A in lung cancer [27], more recent studies demonstrated that activation of retinoic acid receptors correlates with worse prognosis for NSCLC, and supplementation with vitamin A increases the risk of lung cancer in smokers [28, 29]. Our findings demonstrate that retinoic acids may contribute to maintenance of a Treg-mediated immunosuppressive microenvironment during chronic airway inflammation, which could help explain the adverse effects of supplementation of vitamin A on lung cancer.

Despite progress in understanding the biology of COPD and lung cancer, the mechanism by which inflammation regulates tumor formation remains poorly defined. In these studies, we demonstrated that chronic NF-κB-driven airway inflammation promotes epithelial malignancies through alternative activation of alveolar macrophages, resulting in TGFβ/IL-10/retinoic acid-dependent generation and maintenance of Foxp3+ Tregs. Potentially, these molecules and pathways could be targeted to reduce the excessive lung cancer risk in individuals with chronic lung inflammation related to COPD.

## MATERIALS AND METHODS

### Mice

*IKTA* mice (IKKβ Trans-Activated) selectively expressing a constitutively active form of human IKKβ in airway epithelium has been reported previously [11]. We used sex-matched *IKTA* mice and *WT* littermate controls (weighing 20-25 grams) on the original FVB background. To activate transgene expression, mice were provided with 0.1 g/L doxycycline (dox) in drinking water. To induce tumors, urethane was given by a single IP injection (1 g/kg). At the time of sacrifice, lungs were lavaged, perfused, fixed in ice cold Bouin's fixative solution (Sigma-Aldrich) for 24 hours, used for surface tumor number and diameter measurements under a dissecting microscope by at least two experienced readers, blinded to sample identifiers and embedded in paraffin. All mouse experiments were approved by the Vanderbilt University Institutional Animal Care and Utilization Committee.

### Bronchoalveolar lavage (BAL)

BAL fluid was collected by flushing the lungs 3X with 1 ml of serile PBS. Total and differential BAL cell counting was performed as previously described [9].

### Isolation of alveolar macrophages

Macrophages were magnetically enriched from BAL using CD11b microbeads (Miltenyi Biotech) and purified by 2 hour adherence to 6-well cell culture dishes in RPMI-1640 media supplemented with 10% FBS.

### Depletion of macrophages with clodronate

Clodronate (dichloromethylene diphosphonic acid, Sigma-Aldrich, St. Louis, MO, USA) or sterile phosphate buffered saline (PBS)-containing liposomes were prepared as previously described [13]. Liposomes were injected weekly using previously described methodology [13].

### Histology and immunohistochemistry

Lungs were sectioned (5 μm), stained with H&E, and analyzed by a pathologist blinded to the experimental groups for evaluation of tumor and atypical adenomatous hyperplasia (AAH) lesions in three separate sections cut at predetermined depths. To analyze lung emphysematous changes, alveolar septal perimeters were measured by a pathologist blinded to the genotype and treatment group. Morphometrical evaluations were performed using computerized image analyzer system (Image-Pro Express, Media Cybernetics, Inc., Silver Spring, MD). To analyze elastin fibers in lung tissue sections were stained with Verhoeff's Van Gieson staining. For arginase-1 localization, lung sections were immunostained with rabbit anti-arginase-1 antibody (Abcam) and goat anti-rabbit Alexa Fluor 549 secondary antibodies (Life Technologies). For staining of macrophages lung sections were immunostained with rat anti-mouse CD68-FITC antibodies (AbD Serotec).

### Real-time PCR

RNA from alveolar macrophages was isolated using the RNeasy Mini kit (Qiagen, Valencia, CA). DNase-treated samples were subjected to Real-Time PCR using SYBR Green PCR Master Mix (Applied Biosystems). PCR primers used were: CCL3 For: 5′-TGCCCTTGCTGTTCTTCTCT-3′, Rev: 5′—GATGAATTGGCGTGGAATCT-3′; TNFa For: 5′-AAGCCTGTAGCCCACGTCGTA-3′, Rev 5′-GGCA CCACTAGTTGGTTGTCTTTG-3′; iNOS For: 5′-CAGC TGGGCTGTACAAACCTT-3′, Rev 5′-CATTGGAAGTGA AGCGTT-TCG-3′; Ym1 For: 5′-GGGCATACCTTTATCC TGAG-3′, Rev: 5′-CCACTGAAGTCATCC-ATGTC-3′; Fizz1 For: 5′-TCCCAGTGAATACTGATGAGA-3′, Rev: 5′-CCACTCT-GGATCTCCCAAGA-3′; IL-10 For: 5′-ACCTGCTCCACTGCCTTGCT-3′, Rev: 5′-GGTTGCC AAGCCTTATCGGA-3′; RALDH 1 For: 5′-ATGGTTTA GCAGCAGGACTCTTC-3′ and Rev: 5′-CCAGACATC TTGAATCCACCGAA-3′; RALDH 2 For: 5′-GACTT GTA-GCAGCTGTCTTCACT-3′, Rev: 5′-TCACCCATTTC TCTCCCATTTCC-3′; TGFβ For: 5′-CAATACGTCAGAC ATTCGGGAAGC-3′, Rev: 5′-CTGGTAGAGTTCTACGT GTTGCTC-3′; GAPDH For: 5′-TGAGGACCAGGTTGT CTCCT-3′, Rev: 5′-CCCTGTTGCTGTAGCCGTAT-3′. Relative mRNA expression in each sample was normalized to GAPDH and presented using the comparative 2^ΔCt^ method, where ΔCt is Ct (GAPDH) — Ct (target gene).

### Measurement of protein concentration

Total TGFβ and IL-10 were measured in cell-free BAL fluid or whole lung homogenates using a specific ELISA (from R&D Systems and Biolegend respectively) according to manufacturer's instructions.

### Western blot analysis

Nuclear protein was extracted using NE-PER Nuclear and Cytoplasmic Extraction kit (Thermo Fisher Scientific). Western blot analysis was performed with antibodies against NF-κB p65 (Santa Cruz) and nuclear matrix protein - p84 (GeneTex), with the Odyssey infrared system (LI-COR).

### Flow cytometry

Lungs were digested as previously described [10]. Cells were stained with antibodies: CD4-FITC, CD25-APC, FoxP3-PE from e-Bioscience (San Diego, CA, USA), CD4-PerCP-Cy5.5, CD45-APC-Cy7, Gr1-PerCP-Cy5.5, and CD11c-Brilliant Violet 605 from Biolegend (San Diego, CA, USA), CD3-PE-Cy7 and CD8-Alexa Fluor 700 from BD Bioscience (San Diego, CA, USA), CD68-FITC from AbD Serotec (Raleigh, NC, USA) and F4/80 from Life Technologies (Carlsbad, CA, USA). Dead cells were excluded using Live/Dead Fixable Blue Dead Cell Stain kit (Life Technologies, Carlsbad, CA, USA). Cells were analyzed using a LSR II flow cytometer (BD Biosciences, San Diego, CA, USA), and data were analyzed using Flow Jo 7.2 software (Tree Star, Ashland, OR, USA).

### Co-culture of T cells with macrophages

Naive CD4+ T cells from spleens of naive FVB were magnetically purified using microbeads (Miltenyi Biotec), stimulated with 1ug/ml of either CD3/CD28 or allogeneic bone marrow-derived dendritic cells (DC) from C57Bl/6 mice and cultured alone or with alveolar macrophages from WT or IKTA mice collected as described above after 3 weeks of continuous treatment of mice with dox (0.1 g/L). Allogeneic bone marrow-derived dendritic cells (DC) were obtained as described previously [10]. To investigate contribution of TGFβ, IL-10 and retinoic acids in generation of Foxp3+ Tregs by alveolar macrophages, cells were co-cultured in the presence of pan-TGFβ neutralizing antibodies (10 μg/ml, clone 1D11, Genzyme Corp) [30], anti-IL-10 antibodies (10 ug/ml, clone JES5-2A5, Biolegend) or IgG1 isotype control antibodies (Biolegend, San Diego, CA, USA) or a RALDH inhibitor, 4-(diethylamino)benzaldehyde (DEAB; 15 μM, Sigma-Aldrich) [20], added to culture media at day 0. After 4 days of co-culture, cells were stained with CD4 and CD25 antibodies, permeabilized, processed for intracellular staining using anti-FoxP3 antibodies and analyzed by flow cytometry. Dead cells were excluded using a Live/Dead Fixable Blue Dead Cell Stain Kit (Life Technologies). For assessment of proliferation, T cells were labeled with CFSE (5 μM; Life Technologies) prior to use in co-culture experiments and proliferation was analyzed using flow cytometry based on CFSE dilution within CD4+/Foxp3-^−^ lymphocytes.

### Statistical analysis

For studies comparing differences between two groups, we used unpaired Student's t-tests. For differences between more than two groups we used one-way ANOVA with an appropriate post-test. Values are presented as mean ± SEM. P < 0.05 was considered statistically significant.
